# Re‐emerging role of macroscopic appearance in treatment strategy for gastric cancer

**DOI:** 10.1002/ags3.12218

**Published:** 2018-10-19

**Authors:** Kei Hosoda, Masahiko Watanabe, Keishi Yamashita

**Affiliations:** ^1^ Department of Surgery Kitasato University School of Medicine Sagamihara Japan; ^2^ Division of Advanced Surgical Oncology, Research and Development Center for New Medical Frontiers Kitasato University School of Medicine Sagamihara Japan

**Keywords:** gastric cancer, macroscopic appearance, precision medicine, therapeutic strategy

## Abstract

Pathological outcomes are definitely the most important prognostic factors in gastric cancer, but they can be obtained only after surgical resection. Use of preoperative adjuvant chemotherapy is becoming widespread for aggressive human cancer, so clinical factors such as macroscopic features are important as they are highly predictive for patient prognosis. In gastric cancer, the macroscopic type represents a distinct prognosis; Type 0 represents early gastric cancer with excellent prognosis, but, among advanced tumors, giant Type III and Type IV tumors have a dismal prognosis. Japan Clinical Oncology Group (JCOG) Stomach Cancer Study Group adopted macroscopic features as high‐risk entities in clinical trials. It makes sense for risk classification to use macroscopic phenotypes because The Cancer Genome Atlas (TCGA) Network has lately subcategorized different histologies associated with specific macroscopic types by the molecular features of the whole genome. Dismal prognosis of Type IV gastric cancer is notorious, but similar prognosis was seen in giant Type III gastric cancer defined as 8 cm or beyond, both of which are unique for their propensity of peritoneal dissemination. In this review, clinical relevance including prognosis of such macroscopic high‐risk features will be separately debated in the context of precision medicine and updated prognostic outcomes will be presented under the present standard therapy of curative surgery followed by postoperative S‐1 chemotherapy. Moreover, promising emerging novel therapeutic strategies including trimodal potent regimens or intraperitoneal chemotherapy will be described for such aggressive gastric cancer.

## 1 INTRODUCTION

Gastric cancer is the fifth most common malignancy and the third leading cause of cancer‐related death worldwide.^1^ Advanced gastric cancer with depth of pT2 or greater continues to show unsatisfactory survival outcomes despite progress in multimodal therapy.

**Figure 1:**
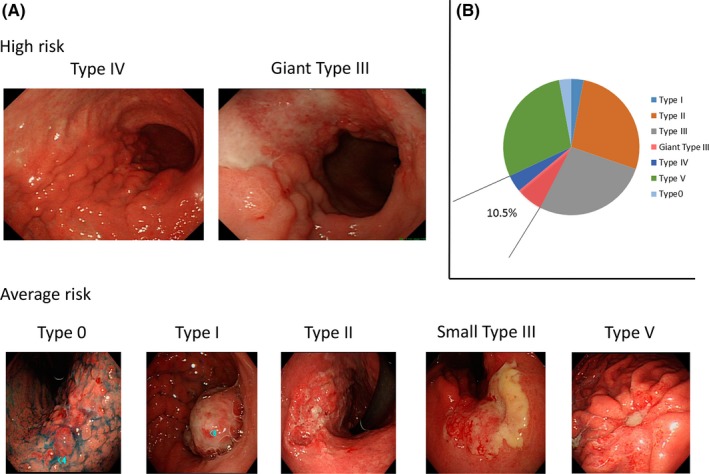
A, Representative gastroendoscopy images of advanced gastric cancer by macroscopic classification. Upper panels include high‐risk macroscopic features of Type IV (left) and giant Type III (right). Lower panels include average‐risk macroscopic features of Type 0, Type I, Type II, small Type III, and Type V (in order from left to right). B, Rate of each macroscopic feature in pathological stage II/III advanced gastric cancer in our own experience. High‐risk macroscopic features (Type IV and giant Type III) are seen in 10.5% as shown in this figure

Macroscopic appearance of gastric cancer is defined in the Japanese classification of gastric carcinoma^2^ in reference to the Borrmann's classification as follows: Type 0 (superficial), typical of T1 tumors; Type I (mass), polypoid tumors sharply demarcated from the surrounding mucosa; Type II (ulcerative), ulcerated tumors with raised margins surrounded by a thickened gastric wall with clear margins; Type III (infiltrative ulcerative), ulcerated tumors with raised margins surrounded by a thickened gastric wall without clear margins; Type IV (diffuse infiltrative), tumors without marked ulceration or raised margins, the gastric wall is thickened and indurated and the margin is unclear; Type V (unclassifiable), tumors that cannot be classified into any of the above types. Regarding Type V, we classified 0‐IIc‐like advanced type tumors into Type V tumors.^3^ Therefore, in the present study, we used the term “Type V” to indicate unclassifiable tumors plus 0‐IIc‐like advanced type tumors (Figure 1A).

The relationship between macroscopic features and prognosis was determined more than 30 years ago.^4^ However, multivariate analyses sometimes prevented independent significance of macroscopic features for the prognosis of gastric cancer patients,^5^, ^6^, ^7^ whereas pathological factors such as tumor depth or lymph node metastasis as well as distant metastasis were almost always selected as independent prognostic factors. Therefore, gastric cancer staging which should accurately predict the prognosis of patients is defined by tumor depth, lymph node metastasis, and distant metastasis.

In Japan, therapeutic strategy for gastric cancer is different according to pathological stage (stage I, stage II/III, stage IV) and, in contrast with the West, preoperative adjuvant chemotherapy is not popular at present. Since the results of the Adjuvant Chemotherapy Trial of TS‐1 for Gastric Cancer (ACTS‐GC) trial were published, postoperative chemotherapy is usually given for pathological stage II/III gastric cancer.^8^, ^9^ However, the prognosis of patients with pathological stage III gastric cancer is so unsatisfactory, even after S‐1 adjuvant chemotherapy, that a more potent chemotherapeutic regimen is needed for such patients. In contrast, performance status of gastrectomized patients is so poor that a potent regimen such as triplet chemotherapy cannot be given. Thus, in practice or in clinical trials, preoperative adjuvant chemotherapy has recently been conducted in Japan.^10^, ^11^, ^12^ Therefore, prognostic factors independent of pathological stage have been helpful for determining the therapeutic strategy, and age and macroscopic features were finally proven to be the most potent independent prognostic factors among preoperatively obtainable ones in advanced gastric cancer requiring gastrectomy.^13^

However, elderly status results in mandatory and compromised therapeutic strategies in terms of lymph node dissection extent, indication for adjuvant chemotherapy, and execution of potent and effective chemotherapy after recurrences, so prognosis of elderly gastric cancer patients rather reflects therapeutic intensity than tumor aggressiveness from a prognostic point of view. It may not represent aggressive intrinsic characteristics of the tumor.

In contrast, macroscopic features have sometimes been reported to be a prognostic factor independent of stage,^3^, ^13^, ^14^, ^15^, ^16^ and this clinical factor is likely to represent tumor aggressiveness. Macroscopic features long denied as a prognostic factor have re‐emerged as crucial for determining therapeutic strategy. Macroscopic features are clearly correlated with histological differentiation, and the differential histology harboring specific macroscopic features has been subcategorized by the molecular features of the whole genome,^17^ suggesting that, in gastric cancer, macroscopic classification of gastric cancer might be essential for precision medicine linked to specific high‐efficacy molecular targeted therapy. Importantly, Japan Clinical Oncology Group (JCOG) considers giant Type III and Type IV tumors as such dismal prognostic phenotypes that it has defined them as high‐risk gastric cancer, and promising new therapeutic strategies are rigorously being developed for optimized treatments in clinical trials to improve survival outcomes.^10^, ^18^

In the present article, we reviewed the re‐emerging clinical relevance of the macroscopic appearance of gastric cancer in the context of rigorous investigation of prognosis and debate the best optimized treatment strategy in the era of precision medicine.

## 2 PROGNOSIS OF GASTRIC CANCER ACCORDING TO MACROSCOPIC CLASSIFICATION

**Figure 2:**
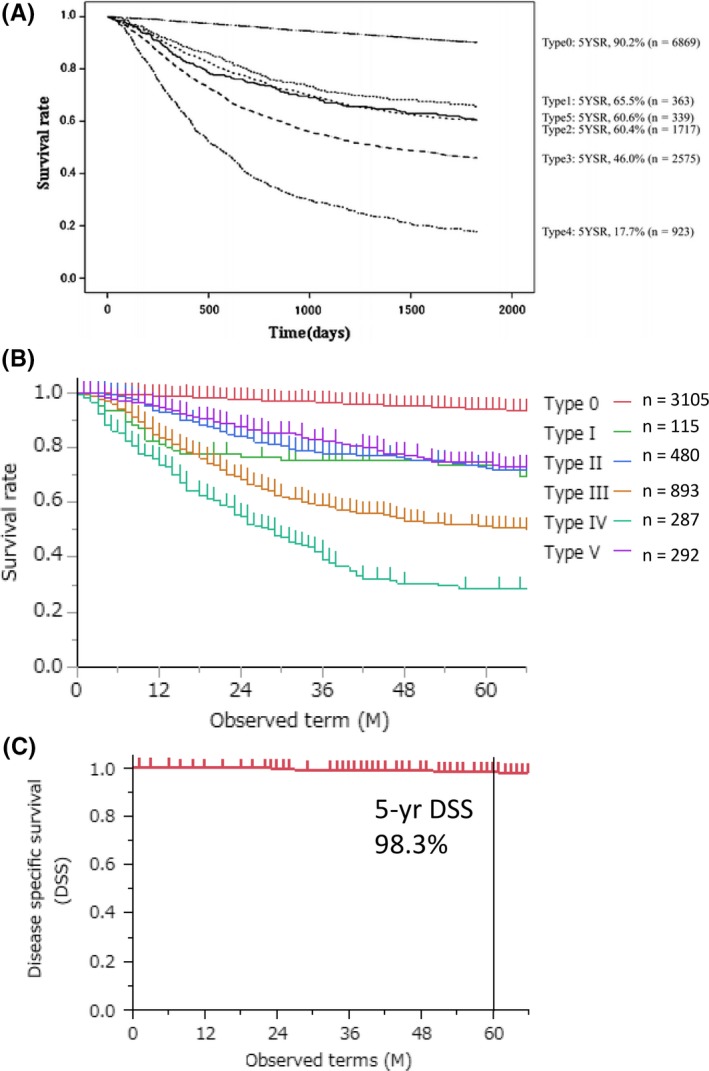
Overall survival of patients with gastric cancer from (A) nationwide registry of gastric cancer and (B) our experience of 5172 gastric cancer patients according to Borrmann's macroscopic features. (C) Disease‐specific survival of 491 cT1 gastric cancer patients

In 2002, the nationwide registry of gastric cancer showed prognosis according to macroscopic features^19^ (Figure 2A). This result is very similar to our single institute experience between 1971 and 2012^14^ (Figure 2B). Macroscopic Type 0 tumor shows excellent prognosis with 5‐year overall survival (OS) beyond 90%. Among advanced gastric cancer (pT2 or beyond), macroscopic types I/II/V showed relatively good prognosis with 5‐year OS between 60% and 70%, and they could be designated as conventional (average‐risk) advanced gastric cancer. In contrast, macroscopic Type III and Type IV tumors showed dismal prognosis as compared to conventional advanced gastric cancer, and they could be designated as high‐risk advanced gastric cancer. For Type III tumors, size of 8 cm can stratify their prognosis into giant Type III and otherwise (small) Type III, which correspond to high‐risk and average‐risk gastric cancer, respectively. Sasako et al first advocated a cut‐off size of 8 cm.^20^ They reported that 5‐year survival rates of patients with Borrmann Type III gastric cancer with tumor size >8 cm and <12 cm and that of >12 cm were 20.3% and 0%, respectively. They concluded that patients with Borrmann Type III gastric cancer with tumor size >8 cm as well as those with Bormann Type IV gastric cancer have dismal prognosis and are candidates for neoadjuvant chemotherapy. According to this report, JCOG recognized Borrmann Type III gastric cancer with tumor size >8 cm and Bormann Type IV gastric cancer as a distinct category. That is why we adopted the size of 8 cm to be the cut‐off for giant Type III. Type IV gastric cancer showed 5‐year OS of 17.6% and 16.6% in the nationwide registry in 2002 and in our institute, respectively. Giant Type III gastric cancer actually showed a similar prognosis to Type IV gastric cancer.^10^

## 3 THERAPEUTIC STRATEGIES ACCORDING TO MACROSCOPIC CLASSIFICATION BASED ON TARGETING RATIONALE

### 3.1 Macroscopic Type 0 cancer

Because of the widespread use of endoscopic screening, over half of Japanese patients who undergo gastric resection for gastric cancer have early stage, that is, macroscopic Type 0 cancer.^19^ Recurrence rate of patients with cStage I gastric cancer undergoing laparoscopic gastrectomy has been reported to be 0%‐2.5%^21^, ^22^, ^23^ (Figure 2C). Even when cT1 tumor turned out to be pT2 or more advanced after surgical resection, the overall survival rate was reported to be as high as 90%.^24^ Survival outcomes of cT1 gastric cancer are so good that we should concentrate on how to improve quality of life (QOL) of patients undergoing surgical resection by carrying out function‐preserving surgery such as proximal gastrectomy,^25^, ^26^ pylorus‐preserving gastrectomy,^27^, ^28^, ^29^ as well as local resection, while securing the same oncological outcomes. For this purpose, sentinel node navigation surgery will be promising for next‐generation therapy in cT1 gastric cancer at present requiring gastrectomy.^30^, ^31^

Features of early‐stage gastric cancer without lymph node metastasis have been well clarified.^32^, ^33^, ^34^ Tumors with very rare incidence of lymphatic metastasis should be resected endoscopically. Japanese gastric cancer treatment guidelines 2014 indicate endoscopic resection for the following tumors rather than gastrectomy: differentiated‐type adenocarcinoma without ulcerative findings [UL(−)] of which depth of invasion is diagnosed as cT1a and diameter is ≤2 cm. In contrast, endoscopic treatment of tumors diagnosed as cT1a and (i) of differentiated‐type, UL(−), but >2 cm in diameter; or (ii) of differentiated‐type, UL(+), and ≤3 cm in diameter; or (iii) of undifferentiated‐type, UL(−), and ≤2 cm in diameter has been considered as investigational.^35^ In the JCOG0607 study, however, endoscopic submucosal dissection (ESD) for cT1a and (i) of differentiated‐type, UL(−), but >2 cm in diameter; or (ii) of differentiated‐type, UL(+), and ≤3 cm in diameter achieved a 5‐year overall survival rate of 97.0%, which was higher than the expected threshold of 86.1%.^36^ Therefore, instead of gastrectomy, indication for endoscopic treatment is planned to be expanded in the next treatment guidelines.

### 3.2 Macroscopic Type I, II, and V cancers

In advanced (cT2/pT2 or deeper) gastric cancer, macroscopic appearance was proven to be a simple and important indicator of prognosis^3^ (Table 1). Macroscopic features of types I, II, and V were a better prognostic indicator than other types independent of tumor stage, and they could be designated as conventional advanced gastric cancer differentially from Type III/IV advanced gastric cancer (Figure 2A,B). In fact, in our experience, prognosis of patients with macroscopic features of types I, II, and V was slightly better than in those with S‐1 adjuvant chemotherapy in the ACTS‐GC trial with a 5‐year overall survival rate of 76.6% and 71.7%, respectively.^3^, ^9^ All patients with types I, II, and V in our experience received adjuvant chemotherapy with S‐1. Currently, D2 gastrectomy with postoperative S‐1 is the standard treatment strategy for this type of gastric cancer. More recently, the JACCRO GC‐07 trial showed that adjuvant S‐1 plus docetaxel is superior to adjuvant S‐1 monotherapy after D2 gastrectomy for pStage III gastric cancer in terms of survival outcomes.^40^ When this treatment strategy of adjuvant S‐1 plus docetaxel after D2 gastrectomy is adopted, prognosis of patients with macroscopic features of types I, II, and V will improve further.

**Table 1:** Previous reports concerning survival outcomes by each macroscopic type

First author/Ref	Year	Macroscopic type	n (%)	5‐y OS rate (%)	*P* value
Yamashita^3^	2017	Others/giant III or IV	154 (90)/18 (10)	76.6/34.0	<0.0001
Huang^15^	2016	Others/IV	1487 (92)/135 (8)	45.3/19.7	<0.0001
Yamashita^14^	2015	IV	287	16.0	NA
Yamashita^13^	2014	I or II or V/III/IV	80 (34)/106 (46)/46 (20)	50^a^/20^a^/5^a^	<0.0001
Noda^37^	2011	Others/IV	1300 (88)/174 (12)	27.7/6.3	0.023
Li^16^	2009	I or II/III/IV	1115 (28)/2334 (59)/517 (13)	67.7/55.2/31.8	<0.001
Msika^38^	2000	I/II/III/IV/Unknown	45 (8)/130 (23)/308 (55)/59 (11)/19 (3)	43.8/40.4/28.9/18.1/Unknown	<0.0001
Kinugasa^39^	1997	IV	73	31.4	NA

NA, not applicable; OS, overall survival.

a Disease‐specific survival.

Among these macroscopic types showing better prognosis, Type I gastric cancer is reported to be relatively rare accounting for 2.2%‐8% of all resectable gastric cancer.^15^, ^16^, ^38^, ^41^ Its detailed prognostic features remain unclear because few prognostic analyses have been carried out. We recently investigated prognostic factors in Type I gastric cancer and reported that patients with a high risk for recurrence had diffuse‐type histology with robust lymphatic invasion.^41^ Recurrence pattern of Type I gastric cancer with intestinal‐type histology is unique as this type of gastric cancer is prone to metastasize to the liver such as colorectal cancer (CRC). Type I gastric cancer showed relatively good prognosis as compared with Type III or IV. Thus, it may be wise to select a safer operation and postoperative adjuvant strategy expecting better QOL.

Type II gastric cancer also showed CRC‐like phenotype with regard to recurrence patterns, where liver metastasis is again the most dominant.^42^ Type I/II gastric cancers are uniquely prone to intestinal‐type histology, whereas Type III/IV gastric cancers mainly had diffuse‐type histology.^13^ Intestinal‐type gastric cancer is composed of Epstein‐Barr virus associated, microsatellite instability associated, and chromosomal instability (CIN) gastric cancer, and the former two were composed of relatively minor portions of intestinal‐type gastric cancer showing good prognosis.^17^ In contrast, CIN gastric cancer is tightly associated with p53 mutation.^17^

**Figure 3:**
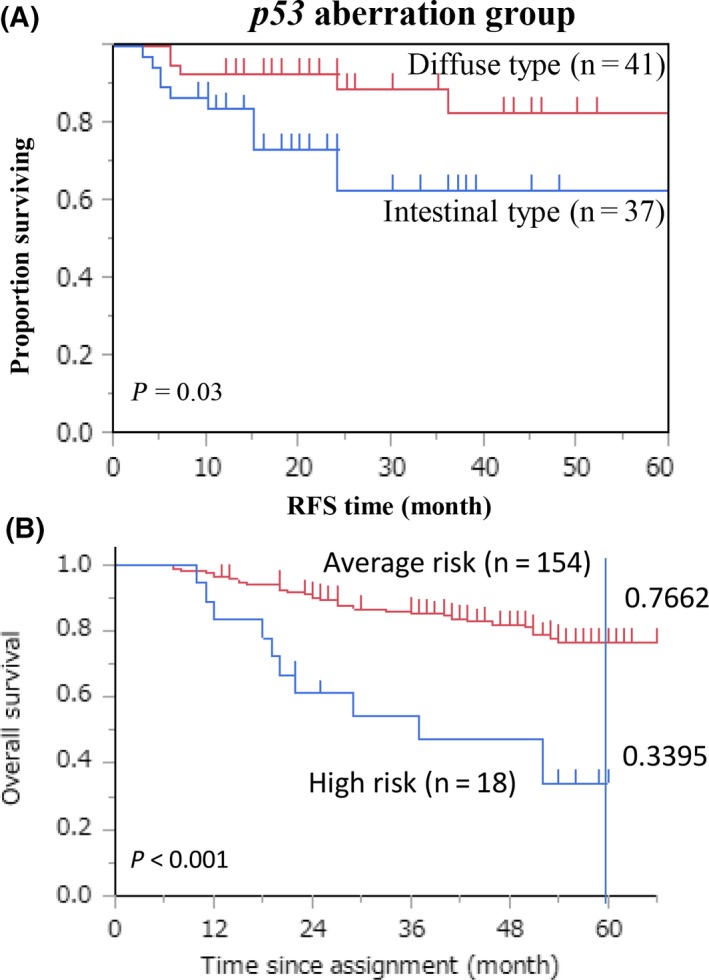
A, Survival curves for intestinal type were compared with those of diffuse‐type gastric cancer with pStage II/III in the p53 aberration group. B, Survival curves according to macroscopic features for the high‐risk group (Type IV and giant Type III group) and the average‐risk group (otherwise Type 0, Type I, Type II, small Type III, and Type V group). Five‐year survival is shown

We identified cancer‐specific methylated genes which are in the *p53* tumor suppressor pathway^43^, ^45^ and ablated the *p53* pathway in tumor tissues in an epigenetic way together with wild‐type *p53*. The identified genes were *PGP9.5*,* NMDAR2B*, and *CCNA1* which were exclusively methylated in primary tumors with no *p53* mutation. Gastric cancer patients were classified into three categories based on the *p53* mutation status as well as on the DNA methylation status of *PGP9.5*,* NMDAR2B* and *CCNA1*. These three categories were *p53* mutant, *p53* wild type with super‐high methylation (SHM) of the above three *p53* pathway genes (*p53* WT/SHM), and *p53* wild type without SHM (p53 WT without SHM). We further designated the p53 mutant plus the p53 WT/SHM groups as the p53 aberration group, and p53 WT without SHM was designated as the p53 no‐aberration group. Although no difference of prognosis between diffuse‐type and intestinal‐type gastric cancer was found in the p53 no‐aberration group, intestinal‐type gastric cancer showed poorer prognosis than diffuse‐type cancer in the p53 aberration group^46^ (Figure 3A).

From such a prognostic point of view, CIN gastric cancer, characterized by p53 mutation, is the most concerning problem regarding the prognosis of intestinal‐type gastric cancer. Intriguingly, a recent report showed that p53 mutation is highly correlated with liver metastasis in gastric cancer,^47^ so CIN gastric cancer deserves attention to target gain of function of the p53 mutation in intestinal‐type gastric cancer beyond the standard treatments.^48^

In contrast, Type V (0‐IIc‐like advanced) gastric cancer largely represents an earlier stage of either Type II or Type III gastric cancer, so it may be composed of both distinct histological phenotypes of gastric cancer with intestinal‐ and diffuse‐type histology. As its prognosis is similar to Type I/II gastric cancer, it should be clinically included into conventional gastric cancer. There have been no detailed reports regarding prognosis of Type V gastric cancer.

### 3.3 Macroscopic types III and IV cancer

Type III is the most frequent macroscopic type of curable advanced gastric cancer (Figure 1B), whereas Type IV is the most disastrous type of gastric cancer from a prognostic point of view. We previously analyzed approximately 5000 patients with gastric cancer who underwent gastrectomy and concluded that Type IV gastric cancer accounted for 5.5% of the patients and was associated with the most dismal prognosis with a 5‐year overall survival rate of 16%.^14^ However, Type III gastric cancer accounted for 17% of patients with a 5‐year overall survival rate of 34% in total cases,^13^ and 59% when peritoneal lavage cytology test was negative (CY0).^49^ In Type III gastric cancer, positive peritoneal lavage cytology test (CY1) was proven to be the strongest independent poor prognostic factor with a hazard ratio of 2.37, while tumor size was also a potent prognostic factor among those with CY0.^49^ Allowing for these prognostic features, in JCOG Stomach Cancer Study Group, giant Type III and Type IV gastric cancer is believed to be a high‐risk gastric cancer among resectable gastric cancer.

Intriguingly, both giant Type III and Type IV gastric cancer (high‐risk cancer) actually showed poorer prognosis than other gastric cancer (average‐risk cancer) even in patients with pStage II/III who underwent current standard treatments (curative surgery followed by S‐1 adjuvant chemotherapy)^3^(Figure 3B). Dismal prognosis of gastric cancer of Type III/IV macroscopic appearance is largely explained by treatment failure of peritoneal dissemination, and neoadjuvant chemotherapy may be promising to improve prognosis of such high‐risk gastric cancer, because perioperative chemotherapy of epirubicin/cisplatin/5‐FU (ECF) can improve the dismal prognosis of gastric cancer in the western world.^50^ Recently, chemoradiotherapy after preoperative chemotherapy and surgery for resectable gastric cancer did not improve overall survival compared with perioperative chemotherapy and surgery.^51^ Therefore, benefits of the addition of radiation to perioperative chemotherapy have yet to be clarified.

Among Type III/IV gastric cancer, Type III and young Type IV had the same survival prognosis that was much better than elderly Type IV gastric cancer.^13^ Elderly Type IV had the most dismal prognosis in resectable gastric cancer, which raises a question as to whether or not patients with this type of cancer should undergo surgery.^52^ Elderly patients do not usually undergo standard therapy such as D2 gastrectomy, standard dose of postoperative S‐1 therapy, and standard chemotherapy of cisplatin/S‐1 (CS) for recurrent disease. These therapeutic factors may affect the poor prognosis of elderly Type IV gastric cancer. However, importantly, peritoneal immunity declines with age.^53^ If immunity is involved in the dismal prognosis of Type IV elderly gastric cancer, an immune checkpoint inhibitor such as nivolumab is a potential candidate to improve prognosis in elderly Type IV gastric cancer.

### 3.4 Potent chemotherapy for high‐risk gastric cancer

Japan Clinical Oncology Group considered neoadjuvant chemotherapy of CS to be a promising therapeutic strategy. In a phase II study (JCOG0210), CS combination chemotherapy provided relatively better survival with a 3‐year survival rate of 24.5% and a median survival term of 17.3 months.^10^ A phase III study (JCOG0501) comparing this preoperative chemotherapy followed by D2 surgery with surgery alone, both of which were followed by postoperative S‐1 adjuvant chemotherapy for 1 year, finished patients’ registration and provided a discouraging result with no additional benefit of neoadjuvant chemotherapy with CS.^11^, ^18^ This result indicated that, at present, there is no indication for neoadjuvant therapy even in high‐risk advanced gastric cancer.

A more potent chemotherapeutic regimen of docetaxel/cisplatin/S‐1 (DCS) was developed in metastatic gastric cancer and achieved a response rate of 81%.^54^ Actually, this result encouraged us to carry out conversion surgery for initially unresectable gastric cancer.^55^ Regrettably, a JCOG1002 study investigating preoperative chemotherapy of DCS for gastric cancer with extensive lymph node metastasis (bulky N) failed to achieve the expected response rate of 80%,^12^ but preoperative DCS chemotherapy was not applied to giant Type III or Type IV. Thus, the KDOG1001 trial is being conducted to validate the clinical effect of DCS neoadjuvant chemotherapy in aggressive gastric cancer including giant Type III and Type IV. Registration has already been completed, and the results are awaited (UMIN 000003642).

Of the patients undergoing conversion surgery, Type III or IV cancer accounted for 87%.^55^ In the integrated analysis of these patients, Type IV cancer was selected as an independent prognostic factor (data not shown). The fact that Type IV cancer has a dismal prognosis and tends to recur at the peritoneum even after aggressive chemotherapy caused us to develop another therapeutic strategy such as the recently emerging i.p. chemotherapy together with i.v. chemotherapy.^56^, ^57^

### 3.5 Therapeutic rationale for peritoneal dissemination in gastric cancer

Peritoneal lavage cytology test did not independently affect overall survival in Type IV gastric cancer.^14^ In Type IV cancer, there may be a potential scattering of cancer cells even in the CY0 status. Hence, we must understand the potential for peritoneal residual disease in all type IV gastric cancer. In fact, detection of promotor methylation of the *CDO1* gene, which is involved in cysteine biology and is thought to be a cancer‐specific DNA marker gene, was able to predict peritoneal metastasis more sensitively than the conventional cytology test, especially for Type III/IV gastric cancer.^58^

In terms of cytology‐positive gastric cancers, we showed that absence of peritoneal dissemination can predict long‐term survival of patients with advanced gastric cancer with a positive cytology test and long‐term postoperative adjuvant therapy with S‐1 was required for survival of patients with CY1 in the absence of peritoneal dissemination over 5 years.^14^, ^59^, ^60^ A recent systematic review also showed that the use of S‐1 monotherapy was associated with a significant survival benefit in CY1 patients (HR 0.48; 95% CI 0.32‐0.70; *P* = 0.0002).^61^ Although randomized controlled trials are needed, S‐1 monotherapy, due to its easy feasibility, may be very promising for gastric cancer with the unresectable factor of cytology positive alone, and this theoretical rationale is applicable to Type III/IV gastric cancer.

Intraoperative i.p. chemotherapy with adjuvant chemotherapy showed a trend toward improvement in overall survival (HR 0.70; 95% CI 0.47‐1.04; *P* = 0.08).^61^ Ishigami et al. conducted a phase III trial comparing i.p. and i.v. paclitaxel plus S‐1 (IP) versus cisplatin plus S‐1 (SP) in patients with peritoneal metastasis.^62^ Although this trial failed to show statistical superiority of intraperitoneal paclitaxel plus systemic chemotherapy, the 3‐year overall survival rate was 21.9% (95% CI, 14.9%‐29.9%) in the IP arm and 6.0% (95% CI, 1.6%‐14.9%) in the SP arm. According to these promising results, adjuvant i.p. chemotherapy with systemic chemotherapy is being planned for resectable Type IV gastric cancer.

## 4 CONCLUSIONS

The present review summarizes the association between macroscopic appearance as well as histological phenotypes to oncological outcomes. In type 0 gastric cancer, survival outcomes are so much better that the extent of cancer resection will become more and more limited to preserve the function of the stomach and maintain the QOL of patients. Indication for endoscopic resection would be expanded and partial resection using sentinel lymph node navigation would be applied to this type of cancer. In stage II/III advanced gastric cancer, treatment strategy will be separately discussed between average‐risk cancer and high‐risk cancer. In average‐risk cancer, adjuvant chemotherapy with DS in addition to adjuvant S‐1 monotherapy will improve the survival outcomes of patients. In high‐risk cancer, which is determined according to the gross appearance of the tumor, development of an effective neoadjuvant chemotherapeutic regimen is still awaited. Average‐risk gastric cancer is composed of Type I, Type II, small Type III, and Type V and has heterogeneous molecular aberrations. Among these, Type I and Type II behave like CRC and are likely to metastasize to the liver. For this type of cancer, a new therapeutic strategy considering p53 aberration might be needed to improve survival outcomes. High‐risk gastric cancer is likely to metastasize to the peritoneum. For the development of suppressing peritoneal dissemination in the future, understanding the molecular pathology of peritoneal dissemination is important. In stage IV gastric cancer, at the present time, chemotherapeutic regimens are evolving to achieve better survival without considering gross appearance of the tumor. Although precision medicine using molecular targeting agents is entering clinical practice, the simple prognostic indicator of macroscopic appearance still has immense importance in selecting the appropriate treatment for gastric cancer.

## DISCLOSURE

Authors declare no conflicts of interest for this article.
